# Diabetes in Argentina: cost and management of diabetes and its complications and challenges for health policy

**DOI:** 10.1186/1744-8603-9-54

**Published:** 2013-10-29

**Authors:** Joaquín E Caporale, Jorge F Elgart, Juan J Gagliardino

**Affiliations:** 1 CENEXA – Centro de Endocrinología Experimental y Aplicada (UNLP – CONICET La Plata, PAHO/WHO Collaborating Centre for Diabetes), Facultad de Ciencias Médicas UNLP, La Plata, Argentina

**Keywords:** Diabetes, Costs, Argentina, Prevalence, Diabetes management

## Abstract

**Background:**

Diabetes is an expensive disease in Argentina as well as worldwide, and its prevalence is continuously rising affecting the quality of life of people with the disease and their life expectancy. It also imposes a heavy burden to the national health care budget and on the economy in the form of productivity losses.

**Aims:**

To review and discuss a) the reported evidence on diabetes prevalence, the degree of control, the cost of care and outcomes, b) available strategies to decrease the health and economic disease burden, and c) how the disease fits in the Argentinian health care system and policy. Finally, to propose evidence-based policy options to reduce the burden of diabetes, both from an epidemiological as well as an economic perspective, on the Argentinian society. The evidence presented is expected to help the local authorities to develop and implement effective diabetes care programmes.

**Methodology:**

A comprehensive literature review was performed using databases such as MEDLINE, EMBASE and LILACS (Latin American and Caribbean Health Sciences). Literature published from 1980 to 2011 was included. This information was complemented with grey literature, including data from national and provincial official sources, personal communications and contacts with health authorities and diabetes experts in Argentina.

**Results:**

Overall diabetes prevalence increased from 8.4% in 2005 to 9.6% 2009 at national level. In 2009, diabetes was the seventh leading cause of death with a mortality rate of 19.2 per 100,000 inhabitants, and it accounted for 1,328,802 DALYs lost in the adult population, mainly affecting women aged over fifty. The *per capita* hospitalisation cost for people with diabetes was significantly higher than for people without the disease, US$ 1,628 vs. US$ 833 in 2004. Evidence shows that implementation of combined educative interventions improved quality of care and outcomes, decreased treatment costs and optimised the use of economic resources.

**Conclusions:**

Based on the evidence reviewed, we believe that the implementation of structured health care programmes including diabetes education at every level, could improve quality of care as well as its clinical, metabolic and economic outcomes. If implemented across the country, these programmes could decrease the disease burden and optimise the use of human and economic resources.

## Background

Diabetes is an expensive disease in Argentina as well as worldwide, and its prevalence is continuously rising [1]. This situation has encouraged us to review published evidence on diabetes prevalence, control, cost of care and outcomes, and to discuss how diabetes fits in the Argentinian health care system and policy. The Argentinian health care system includes three independent sectors: the public, the social security and the private (pre-paid) sectors [2]. The public sector is mainly financed through taxes and provides universal access to free health care to nearly 16 million people (mostly unemployed and low-income population who are not insured through social security or private sector) through primary care units and hospitals with different levels of complexity [2]. It includes different kinds of disease management programmes for the ambulatory treatment of chronic diseases with free supply of drugs through public entities; however, not all chronic diseases are fully covered. Diabetic patients can access insulin, some oral drugs and a limited number of strips for self-monitoring blood glucose (SMBG) free of charge as part of public health insurance. The social security sector comprises more than 300 institutions that depend on provincial governments and different trade unions. They are organised at national and sub-national level and cover nearly 17 million people. The level of health coverage is fixed by law in the so-called Mandatory Health Programme (MHP) [3]. This sector is financed by a fixed compulsory contribution (a percentage of the salary) made by both employee and employer (3% and 6%, respectively). Finally, the private sector is financed through prepaid medical plans; it covers about 3.2 million people and operates in a similar manner as the social security sector. The magnitude of coverage depends on the plan selected by the individual: the lowest-cost plan considers the MHP as the standard reference for the minimum level of coverage required [2].

This study aims to review the status of diabetes management in Argentina, trying to identify the key challenges that the country needs to address and the potential strategies available to reduce the socioeconomic disease burden. We hope that this analysis can help local authorities in Argentina to develop and implement effective diabetes care programmes with the aim of reducing the health and socioeconomic burden this disease continues to inflict to the country.

## Methods

We performed a comprehensive literature review in MEDLINE, EMBASE and LILACS (Latin American and Caribbean Health Sciences) for the period 1980–2011, using the following key words: *diabetes in Argentina, diabetes cost of care, diabetes cost of management, cost of diabetes complications, diabetes health policy.* Abstracts - either in English or Spanish - from the identified references were collected and screened by the authors for inclusion in the study. Population based publications on diabetes prevalence, incidence, social and economic impact of the disease with careful statistical data analysis were included in the revision while those related to the issue but having no these characteristics were excluded. Full papers of the selected abstract were obtained and carefully analysed by the authors. The literature review was complemented with personal communications and contacts with national and provincial health authorities and diabetes experts in Argentina in an attempt to include unpublished evidence and policies. The information collected was then reviewed and summarised independently by the authors and thereafter the selected material was analysed and used to prepare the final manuscript.

## Results

### Diabetes burden: incidence, prevalence, mortality and disability-adjusted life years (DALYs)

Estimates of diabetes incidence are not precise in Argentina. Prevalence values are more reliable and show an increase from 8.4% to 9.6% between 2005 and 2009 at national level (Table 1). Earlier estimates showed a prevalence of 5% in 1987 [4]. Estimates for 2005 and 2009 are based on patient self-reporting during the first and second National Risk Factor Survey performed by the National Health Ministry [1].

**Table 1:** Prevalence of cardiovascular risk factors

**Variable**	**National risk factor survey**	
	**2005**	**2009**
Overweight (BMI> 25 and <30)	34.4%	35.4%
Obesity (BMI = 30)	14.6%	18.0%
Sedentarism/lack of physical exercise	46.2%	54.9%
Hypertension	34.5%	34.8%
Hypercholesterolemia	27.9%	29.1%
Diabetes	8.4%	9.6%

Source: National Survey of Cardiovascular Risk factors – National Ministry of Health.

Both surveys used a four-stage probabilistic sampling design and the samples were representative of 96% of the adult population living in urban areas (5,000 inhabitants or more) [1]. Pilot testing of the questionnaire found a close relationship between direct measurement of weight, blood pressure, glycaemia and total cholesterol and the corresponding self-reported data. Questionnaire validity was not significantly affected by gender, age or educational level [1,5].

### Mortality

Between 1997 and 2006, the crude diabetes mortality rate rose from 19.6 to 21.3 per 100,000 inhabitants [6]. In fact, in the latest available data (2009), diabetes was the seventh cause of death with a crude mortality rate of 19.2 per 100,000 inhabitants and was responsible for 7,701 deaths in the same year; 66% of these deaths corresponded to people aged 55 years or older, and cardiovascular disease was the main cause of death [7]. However, we believe that, as it occurs in many other countries, diabetes mortality rate is underestimated in Argentina due to inappropriate registration of the disease as a second or third underlying cause of death.

### DALYs

Concerning DALYs, a preliminary analysis carried out by CENEXA using the methodology developed by Murray and Lopez [8] showed that in 2005, diabetes alone (without any chronic complication), together with events of acute myocardial infarction (AMI) and stroke attributable to diabetes, accounted for 1,328,802 DALYs lost in the adult population; 85% of this burden was ascribed to disabilities. These results are explained by the chronic and progressive course of the disease [9,10].

Most of this burden affects women (60%; 793,011 DALYs) and people over 50 years old (52%; 694,694 DALYs). A preliminary sensitivity analysis conducted over r and K indicated that different scenarios (r = 0%, K = 1; r = 3%, K = 0; and r = 3%, K = 1) accounted for 90%, 69% and 60% of the base case recently reported (r = 0%, K = 0), respectively [9,10].

On the other hand, a study performed in 2005 by national health authorities using the Global Burden of Disease (GBD) methodology and including other diseases [11] showed that diabetes accounted for 128,576 DALYs, where the years of life lost (YLL) represented 51% of the total DALYs. However, these results do not match well with the GBD data reported by CENEXA and other authors, where disabilities were the main cause of DALYs [11].

According to the National Study of Disease Burden already mentioned, diabetes was the 9th and 11th cause of YLL for women and men, respectively. The top five conditions were different for men and women, but cardiovascular disease and cancer were the two most common conditions in both groups. Regarding DALYs, diabetes occupied the 6th and 7th place in women and men, respectively [11].

It is not easy to estimate precisely the possible variation of diabetes burden in Argentina over the past decade because the available evidence was obtained using different methodologies, many of which have changed over time. However, since the first and the second National Risk Factor Surveys were performed using the same methodology, they provide a more strong evidence base to support an increase in disease prevalence and we can infer that the diabetes associated burden parallels that growth.

### Diabetes treatment and quality of care

There are two different data sources to assess the quality of diabetes care processes and outcomes in Argentina: the QUALIDIAB [12,13] and the International Diabetes Mellitus Practice Study (IDMPS) [14,15]. QUALIDIAB is a systematic and continuous survey of the quality of care provided to people with diabetes in different Latin American countries. It started as an initiative of DOTA (Declaration of the Americas) and was sustained over time by CENEXA. IDMPS is an international survey of quality of care under "real world conditions" which was implemented and sustained by pharmaceutical manufacturer Sanofi-Aventis. QUALIDIAB and IDMPS studies employ a structured survey form that is randomly provided to endocrinologists/diabetologists and general practitioners in similar proportion. Both registries include demographic characteristics, clinical (body mass index (BMI), blood pressure) and metabolic indicators (HbA1c, total cholesterol, HDL-cholesterol and triglyceride measurements [TG]), performance of preventive processes (monitoring of fundus oculi, feet control) and their outcomes. They also record type of treatment, chronic complications, hospitalisations and coronary risk status for people with diabetes.

The data recorded showed that 80% of the people with Type 2 Diabetes Mellitus (T2DM) are overweight/obese (BMI≥25) [10-13], only 45% of them have HbA1c levels ≤7%, and 24% have systolic blood pressure (SBP)/diastolic blood pressure (DBP) ≤130/80 mm Hg. Furthermore, only around 5% of people with T2DM attain these three therapeutic goals – normal weight (BMI 18.50-24.99), glycaemic and blood pressure control - simultaneously (IDMPS third wave in Argentina, unpublished data). Consequently, more than 66% of this patient population is at high risk of developing chronic complications.

Regarding pharmacological treatment, the IDMPS data showed that 91% of patients with Type 1 Diabetes (T1DM) were treated with insulin and 9% with insulin plus oral glucose lowering drugs (OGLD). On the other hand, 66% of patients with T2DM were treated only with OGLD, 2% with diet and exercise, 10% with insulin and 21% with insulin plus OGLD (Table 2).

**Table 2:** Diabetes type of treatment

**Treatment**	**T1DM (N=206)**	**T2DM (N=646)**
Only OGLD	--	65.8
Only Insulin	90.8	10.5
OGLD + Insulin	9.2	21.4
Diet and Exercise	--	2.3

OGLD: Oral Glucose Lowering Drugs. Data expressed as percentage.

Source: IDMPS unpublished data.

Similarly, a study conducted in 43 health centres in Argentina comprising 1,899 patients with T2DM [16] showed that pharmacological treatment was used in 84.4% of the diabetic participants in this study. Of the total 1,899 T2DM patients, 39.7% received one drug, 35.3% two drugs and 9.4% three drugs. Metformin was the drug most frequently used (67.3%), while 31.9% of patients received insulin.

Within the group of CVRFs, diabetes has a privileged position because there is a national diabetes law that regulates the accessibility to care, drugs and strips for SMBG.

The overall capacity of the health care system to meet patients’ needs depends on the health sector considered. There is a clear overload in the public sector, particularly concerned with the daily number of patients they have to take care and some deficits in drug and strip provision. These characteristics are not observed in the social security and private sectors due to the better adjustment among patients number, physicians and allocated budget. Most provinces have adhered to the National Diabetes Programme (PRONADIA) [17] and are trying to increase their capacity to cope with the real needs for quality care and treatment.

### Complications

The QUALIDIAB registry shows that the Argentinean diabetic population has frequent chronic complications and poor control of hyperglycaemia and the associated CVRFs (Table 3). As it occurs in many countries, cardiovascular diseases are the most prevalent chronic complications [12,13,18-23]. However, microangiopathic complications also represent a heavy burden: diabetes is the main cause of end-stage renal disease, while diabetic retinopathy is the first cause of non-traumatic blindness in the adult population [24].

**Table 3:** Prevalence of diabetes chronic complications according to disease duration

**Complication**	**Diabetes duration (years)**			
	**0 - 5**	**6 - 10**	**11 - 20**	**=20**
Blindness	1.6	2.6	3.8	6.9
AMI	10.1	10.2	16.8	16.1
Stroke	8.2	9.7	10.4	8.1
ESRD	0.3	0.5	1.1	2.6
Amputation	3.8	5.0	9.0	15.7
Postural hypotension	55.9	52.3	50.1	36.5
Angor	29.1	27.1	28.9	24.9
Neuropathy	55.2	61.7	69.0	73.3
Lower limb claudication	25.0	29.8	31.7	36.7

AMI: Acute myocardial infarction. ESRD: End-Stage Renal Disease. Data expressed as percentage. Source: QUALIDIAB Database 2006.

The proportion of patients screened for complications and their risk-factors in the last 12 months are described in Table 4.

**Table 4:** Preventive process performance in the last 12 months

**Parameter**	**T1DM (N = 2,160)**	**T2DM (N = 16,445)**	**Total (N = 18,605)**
Body weight	99	99	99
Height	89	93	92
Blood pressure	96	99	98
HbA1c	61	40	43
FBG	74	83	82
Creatinine	89	51	56
Total cholesterol	71	74	74
HDL-cholesterol	52	61	60
Triglyceride	58	68	67
Micro-albuminuria	28	8	11
Foot exam	39	55	54
Retina control	60	45	46

FBG: Fasting blood glucose. Data expressed as percentage.

Source: QUALIDIAB Database 2006.

Although QUALIDIAB and IDMPS have a good quality data on diabetes control, their representativeness of the whole Argentinean population is limited due to small sample size [12,14].

### Diabetes costs: available evidence on its magnitude

Hospitalisation is the main component of the total direct cost per person of diabetes care [19,21,25]. In 2004, Gagliardino et al. evaluated the characteristics of hospitalised patients and inpatient costs in a cohort of social security sector insurees, with and without diabetes [19] (Table 5). While diabetes represented 6% of all the hospitalisations recorded, its cost accounted for 10.5% of the total inpatient cost.

**Table 5:** Local evidence in cost of diabetes

**Study**	**Evidence**	Cost values*
Olivera et al. (1991) [28]	Total costs for absenteeism and early retirement in a cohort of people with diabetes	US$ 374,000 and US$ 29,929,900, respectively.
Gagliardino et al. (2004) [19]	Hospitalization due to cardiovascular disease in diabetic people vs. non-diabetic people	US$ 1,628 vs. US$ 833
	Hospitalization due to acute vs. chronic complications in diabetic people	US$ 2,096 vs. US$879
	Most expensive causes of hospitalization in diabetic people: cardiac and peripheral vascular events	US$ 2,476 and US$ 2,219, respectively.
Gagliardino et al. (2006) [20]	Direct medical cost per capita of a comprehensive diabetes care programme vs. control group (without structured programme)	US$ 1,733 vs. US$ 2,429
Caporale et al. (2006) [21]	Pre vs. post-hospitalization ambulatory care cost of people with diabetes over the same period of time (6 months)	US$ 904 vs. US$ 798
Caporale et al. (2010) [9]	Additional net annual per capita cost of simulated treatments to avoid hospitalization in diabetic people	US$ 400 to US$ 530
Caporale et al. (2011) [26]	Incremental costs of a public health care programme for people with T2DM without complications	AR$ 1,503 to AR$ 1,141

*Cost data are reported without any intertemporal adjustment.

Cardiovascular diseases were the leading cause of hospitalisation in the two groups studied. The average inpatient cost of a diabetic patient was significantly higher than that of a non-diabetic patient (US$ 1,628 *vs*. US$ 833). Comparable average duration figures were recorded in people with diabetes for acute (6.3 ± 5.2 days) and chronic (7.9 ± 8.3 days) complications associated to the disease. However, the average cost per capita of a complicated diabetes case was significantly higher than that of a non-complicated diabetes case (US$ 2,096 *vs*. US$ 879; P< 0.01).

Cardiac and peripheral vascular events were the most expensive causes of hospitalisation (US$ 2,476 and US$ 2,219, respectively). People with diabetes spent more days in hospital than those without the disease (7.8 *vs.* 4.8 on average; P< 0.01) and stayed more days in intensive care units (ICUs) (3.2 *vs.* 0.7; P< 0.05). Finally, the re-hospitalisation rate of people with diabetes was 5.5 times higher than that of non-diabetic patients (P< 0.01) and was significantly associated with a history of severe episodes of acute (odds ratio: 3.61; 95% CI: 1.11–11.70; P< 0.05) and chronic (odds ratio: 4.26; 95% CI: 1.60–11.29; P< 0.01) complications.

We have also analysed and compared the ambulatory care cost of people with diabetes covered by an Argentinean private health insurer during the pre-and post-hospitalisation period with that from non-hospitalised ones during the same period [21] (Table 5). Cardiovascular diseases were the main cause of hospitalisation (43.1%), with a significantly higher per capita cost compared to any other identified cause (mean ± standard error (SE): US$ 1,673 ± US$ 297; P< 0.05). The total annual direct cost *per capita* of hospitalised patients was higher than that of non-hospitalised ones (US$ 2,908 ± 262 *vs.* 473 ± 10, respectively; P< 0.01). Further, the total post-hospitalisation ambulatory care cost was 12% higher but not significantly different from that of the pre-hospitalisation period (US$ 904 ± US$ 109 vs. US$ 798 ± US$ 15). Related to this issue, Caporale et al. (2011) have shown, using a simulation model, that attainment of target HbA1c values by the provision of appropriate treatment to people with T2DM could avoid future hospitalisation events [26] (Table 5). The additional net annual per capita cost of the simulated treatments ranged from US$ 400 to US$ 530, thus having a reasonable cost-consequence rate from a third payer´s perspective. This preventive policy would simultaneously decrease cardiovascular complications that require high-cost hospitalisation, thus reducing spending on diabetes and its complications. These results together with those obtained in PROPAT (study described in detail later in this article), suggest that intensive treatment of hyperglycaemia and its associated CVRFs may prevent hospitalisation events, thus providing a more cost-effective option than the coverage of hospitalisation and post-hospitalisation ambulatory care.

On the other hand, using a micro-costing approach with a probabilistic sensitivity analysis following Monte Carlo simulation, Caporale et al. estimated the incremental costs of a health care programme for people with T2DM without complications in two Argentinean provinces, Córdoba and Misiones, the first characterised by the relatively higher socioeconomic status of its inhabitants in comparison to the second [27]. For the purpose of the study, a public health payer at sub-national level was chosen, and the comparator was a province without a diabetes programme. The estimated incremental annual health care cost per patient in deterministic terms was AR$ 1,503 and AR$ 1,141 for Córdoba and Misiones respectively. In both provinces, the main component of such cost was SMBG (around 50%), followed by the treatment of hyperglycaemia, dyslipidaemia and hypertension, while human resources represented the lowest one (<5%). Since SMBG was the main determinant of treatment costs, its provision should be carefully regulated to avoid inappropriate use of resources and inequities when implementing insulin treatment (in T2DM), which would demand more frequent monitoring. This local evidence could facilitate the implementation of diabetes programmes at the public health care sub-sector level in provinces/countries with comparable socioeconomic and health care settings. It would also help to optimise resource use.

Regarding productivity costs, Olivera et al. reported that the absenteeism of the people with diabetes but without complications was not significantly higher than the people without the disease (13 *vs*. 9 days) [28]. Conversely, people with diabetes and chronic complications lost a significantly higher number of working days than people without complications (106 *vs.* 13 days). The study also demonstrated that in the province of Buenos Aires, people with diabetes retired earlier from their jobs due to permanent disabilities and all these retirements represent a lost of 11 working years. The corresponding costs for absenteeism and early retirement were US$ 374,000 and US$ 29,929,900, respectively.

All the cost data we report in this paper were not adjusted for inflation. Due to the high levels of volatility characterising many Latin American economies, this may influence an inter-study cost comparisons but does not affect the individual study results here reported.

### Effective strategies to reduce diabetes costs

Some effective strategies to reduce the health and cost burden of diabetes to the Argentinean health system and society have been already successfully implemented at the local level. One example is the implementation of a comprehensive diabetes care programme (PROPAT) in the social security sector from 1998 to 2000, which led to significantly improved clinical and biochemical indicators and higher quality of care for people with T2DM [20]. Additionally, the total annual per capita health care expenditure (including ambulatory diabetes care, drugs, laboratory tests and inpatient) was significantly lower in people in the intervention group than in the control group (US$ 1,733 vs. US$ 2,429; P< 0.01).

Evidence from a structured group education programme for people with T2DM implemented simultaneously in ten Latin American countries (including Argentina), showed improved quality of health care indicators and a decrease by 64% of the cost of drug treatment [29,30]. Further, implementation in a primary care setting, of an intervention that included education of patients and physicians combined with comprehensive health care coverage resulted in long-term (three years) improvement in clinical, metabolic and psychological outcomes at the best cost-effectiveness ratio [31].

These local results seem to suggest that a large scale implementation of diabetes education programmes at all levels of care could decrease the current disease burden and economic cost due to diabetes in Argentina and optimise resource use.

### Health care policy on diabetes

As mentioned before, Argentina has a national programme for the prevention and control of diabetes (PRONADIA), created by Law No. 23.753/89 and regulated by PEN Decree No. 1271/98 [17]. This Programme attempts to engage and commit the provinces to develop and implement sub-national programmes for disease control and treatment. Its main aim is *"to improve the quality and life expectancy of people with diabetes, prevent or decrease chronic complications of this disease and ensure the consequent decline of direct and indirect costs. In order to achieve these aims it promotes the active implementation of a prevention and control programme that prioritises appropriate interventions on hyperglycaemia and the associated CVRFs and its chronic complications. Its role is to coordinate and provide technical support for the programming and implementation of provincial programmes”*. Currently all our provinces have either adopted or adapted the PRONADIA principles, being supported with their own budget (federal health care system). Additionally, the National Health Ministry has also developed guidelines for the prevention, diagnosis and diabetes treatment targets, based on international guidelines and with the participation of different academic, scientific and health care organisations [32]. Although the last edition of the document was in 2009, it has been difficult to assess the degree of its implementation and use.

In 2010, PRONADIA tried to widely implement the QUALIDIAB registry (started in 1999 by CENEXA [12,13]) through the provincial diabetes programmes, to assess and follow up objectively the quality of care provided to people with diabetes and other CVRFs. This system has introduced small changes in the original QUALIDIAB data record in order to use a single data collection form for all the CVRFs. This new form, that includes the impaired glucose tolerance and impaired fasting glucose conditions, is part of a WHO programme to identify people with CVRFs and their treatment. The data recording sheet includes indicators on processes, clinical and metabolic outcomes, drug consumption, hospitalisation events and chronic complications.

As mentioned before, there is a programme for the free provision of drugs at national level (REMEDIAR); it supplies metformin, glibenclamide and human insulin to publicly insured diabetic patients through primary care units and public hospitals all over the country. Additionally, the Superintendencia de Servicios de Salud is the national institution that assess the degree of coverage of care and drug treatment by all institutions of the social security system as well as prepaid ones, as established in the PRONADIA, thus they have to cover 100% of the cost of insulin and traditional oral hypoglycaemic agents as well as up to 300 strips for SMBG/year. The free provision of new drugs and insulin analogues is not automatically granted and their coverage/provision is under an audit system, i.e. physicians have to demonstrate in a written form, how essential is its usage.

## Discussion

The data currently described clearly demonstrate that in Argentina, diabetes care consumes a large amount of resources due to its high prevalence and its association with other CVRFs, along with the presence of chronic complications. These complications increase the cost of care and exert a negative impact on quality of life of people with diabetes, but also affect the economy due to productivity losses and the community because of disability, premature mortality, increased spending on health, disability and early retirement benefits, etc.

The negative impact of the development and progression of chronic complications can be effectively prevented by tight control of hyperglycaemia and the associated CVRFs [33-37]. However, the frequent combination of late diagnosis, inappropriate quality of care provided to people with diabetes and uneven access to care and treatment play against the effectiveness and feasibility of secondary prevention [12,13,18-20,22,25,38-42].

The patients overload affecting the public health sector also conspires against the provision of effective care and the consequent prevention of chronic compliactions. Further, in many cases, the patient does not have 100% medication coverage (which is the case for certain new drugs, but not for human insulin or agents such as metformin or glibenclamide), and the consequent extra out-of-pocket (OOP) expenditure becomes an additional burden for the patient. So far however, we have not been able to objectively measure the real burden of OOP payments on individuals and any socioeconomic disparities in access it may lead to due to lack of data.

Together, all the conditions mentioned above can result in the provision of poor quality care and disparities in access for people with T2DM. As a consequence, many patients do not attain the recommended therapeutic goals and develop chronic complications with the negative consequences mentioned above.

When health care planners and authorities analyse possible solutions to tackle these problems, one of the main suggestions emerging it the need to increase the health care budget. Since most developing countries face economic constraints, this option appears difficult to implement. But if such an increase were feasible, it would not necessarily provide a real solution. In fact, using data from countries with higher health care budgets than Argentina, CENEXA has demonstrated that the quality of care provided was similarly poor in all of them [22]. These findings thus showed that the size of the health budget is not the main constraint for delivering good quality diabetes care and that there is a need to find more efficient interventions to improve health care quality.

Implementation of educational interventions at every level could be a potentially effective solution. However, for such intervention to be successful, reluctance of many health care providers and people with diabetes regarding the importance of the active participation of patients in the control and treatment of their disease, need to be overcome [43]. Further, the favourable impact of educational interventions implemented in Argentina in different health care settings, upon clinical, metabolic, psychological and economic indicators needs to be promoted [20,29-31]. Adopting this concept, the Argentinean National Health Ministry has implemented a long-distance diabetes training programme [24] delivered by the universities of La Plata (Argentina) and Indiana (USA) to improve the knowledge and skills of general practitioners around the country.

Despite the great efforts and resources thus far devoted to improving the quality of care of people with diabetes and secondary prevention, the results so far obtained were really poor. Even though diabetes primary prevention has been effectively achieved in people at high risk of developing the disease in different health systems and in different ethnic populations by adopting a healthier life style [44], this approach has not been tested in Argentina. Consequently, CENEXA is actively working in this direction validating several questionnaires suitable for the identification of populations at high risk of developing diabetes [45,46], and organising a multi-sector diabetes primary prevention pilot study in three cities from the province of Buenos Aires to be launched in 2013.

### Recommendations

**Figure 1:**
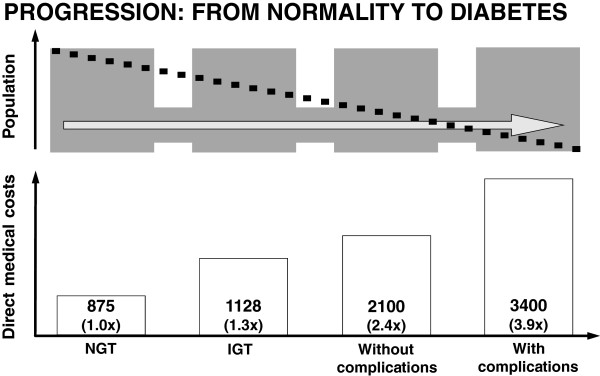
Progression from normal state to diabetes.

How do we envisage the close future activities and the establishment of diabetes health care priorities in Argentina? We did not currently identify any effective long-term programme directly related to either primary or secondary diabetes prevention. We envisage that an improvement in diabetes outcomes in Argentina would require a set of coordinated strategies able to prevent both the transition rate from glucose intolerance to diabetes for people at high risk of developing diabetes (primary prevention) and to prevent the development and progression of chronic diabetes complications (secondary prevention), thus decreasing the high social and economic cost of the disease (Figure 1).

**Figure 2:**
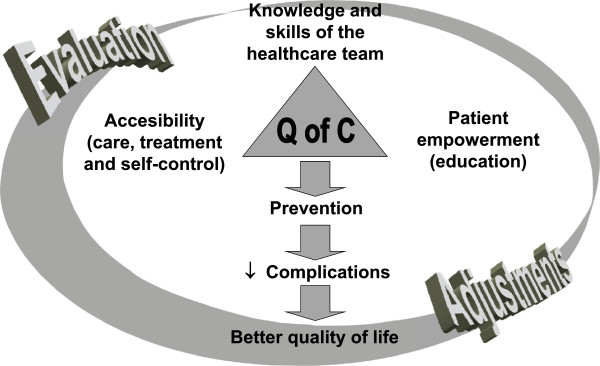
Quality of care synergy.

Such strategies should be mainly based on education programmes implemented at every level of the health care system, including health providers and people with diabetes as well as focussing on improving access to care and treatment (Figure 2).

In brief, we strongly believe that in Argentina the burden of diabetes can be overcome using the approach presented in Figure 2, i.e., implementing education strategies at the level of health care providers and people with diabetes, and also at health care management level (those responsible for health care programme organisation and implementation). A nationwide education programme must be implemented together with a continuous evaluation programme to record successful results and failures that can allow the introduction of changes to optimise outcomes. The evaluation should include not only clinical, metabolic and satisfaction indicators, but also appropriate economic indicators (e.g. direct and indirect costs). The latter can serve to optimise resource allocation based on real needs rather than on transient political demands and pressures.

To increase the effectiveness of the education programme, its design and development should include all the sectors involved in disease management (health care authorities, health care organisations and providers as well as people with diabetes). The public education sector should also be included, since adoption of healthy life style habits must start from an early age, preferably during primary school, when children develop their future habits.

## Competing interests

In the past five years we have not received reimbursements, fees, funding, or salaries from an organization that may in any way gain or lose financially from the publication of this manuscript, either now or in the future. JEC is coordinator at the Unit of Health Economics at CENEXA. JFE is senior investigator at the Unit of Health Economics at CENEXA. JJG is member of the Research Career of the Argentine National Research Council (CONICET) and Director of CENEXA.

## Authors’ contribution

JEC, JFE and JJG designed and performed the local data search, selected and analysed the pertinent data and wrote the manuscript. All authors read and approved the final manuscript.
